# Ammoniation of low-quality roughages for dry-season ruminant feeding: a review

**DOI:** 10.1007/s11250-026-05155-x

**Published:** 2026-06-15

**Authors:** Waheed Idowu, Aureliano José Vieira Pires, Fabio Andrade Teixeira, Grazielle de Carvalho Reis, Geisyane Melo de Queiros, Ionara Souza Machado, Mariana Elmo Fernandes Vilasboas, Tawakalit Remilekun Sani

**Affiliations:** 1 Department of Technology, Rural and Animal Science (DTRA), Southwest Bahia State University (UESB), Itapetinga, Bahia Brazil; 2 Department of Animal Science, Federal University Dutsin-Ma, Dutsin-Ma, Katsina Nigeria; 3 National Animal Production Research Institute, Ahmadu Bello University, Shika-Zaria, Kaduna Nigeria

**Keywords:** Ammoniation, Climate-smart livestock, Digestibility, Dry season, Roughages

## Abstract

Seasonal feed scarcity remains a major constraint to ruminant production in tropical and semi-arid regions, where low-quality crop residues and mature forages dominate dry-season diets. Ammoniation, using anhydrous ammonia or urea-based treatment, represents a practical approach to improving the nutritive value of these resources; however, its mechanistic basis and system-level implications have not been comprehensively synthesized in recent literature. This review integrates evidence published between 2015 and 2025, complemented by foundational studies, to examine the biochemical mechanisms, nutritional responses, and production- and environmental-level outcomes of ammoniation in tropical livestock systems. Ammonia-induced disruption of lignin–carbohydrate linkages enhance fiber accessibility and nitrogen availability, generally resulting in increased intake (10–40%), improved digestibility (5–20% units), and enhanced rumen fermentation. These changes are generally associated with improved animal performance, although responses depend on the synchronization of nitrogen release with fermentable energy supply and overall diet composition. At the system level, improved feed utilization may reduce emission intensity and promote the use of locally available biomass, supporting climate-smart livestock production. However, variability in treatment conditions, potential nitrogen losses, and adoption constraints remain important limitations. Emerging approaches, including integration with biological treatments and digital decision-support tools, offer potential to improve consistency and scalability. Overall, ammoniation remains a relevant strategy for enhancing feed utilization, productivity, and environmental performance in tropical ruminant systems.

## Introduction

Seasonal feed scarcity remains a major constraint to ruminant production in tropical and semi-arid regions, where forage quantity and nutritive value decline sharply during the dry season (Tamboli et al. 2023). Under these conditions, animals rely heavily on crop residues and mature forages characterized by low crude protein (CP) content, high structural fiber, and limited digestibility, resulting in reduced intake, poor body condition, and sub-optimal productivity (Shah et al. 2025; Kumssa 2017).

A range of technologies has been developed to improve the feeding value of low-quality roughages (Asmare 2020). Among these, ammoniation using anhydrous ammonia, aqueous ammonia, or urea-based treatment remains one of the most practical and widely applicable approaches, particularly in smallholder and resource-limited systems. The process enhances nitrogen content and improves fiber degradability through ammonia-induced disruption of lignin–carbohydrate linkages, thereby increasing the accessibility of structural carbohydrates to rumen microorganisms and improving overall digestibility and intake (Leon-Tinoco et al. 2025).

In addition to its nutritional benefits, ammoniation has gained renewed attention within the context of climate-smart livestock production due to its potential to improve feed conversion efficiency, promote the utilization of locally available biomass, and reduce environmentally harmful practices such as open-field burning of crop residues (Adesogan et al. 2025). Emerging perspectives also highlight its relevance in reducing emission intensity at the production-system level, although such outcomes depend on effective management of nitrogen use and feeding strategies.

Although ammoniation has been extensively studied over several decades, recent advances—including improved understanding of treatment kinetics, integration with complementary biological approaches, and the emergence of digital decision-support tools—have not been comprehensively synthesized within a single framework (Marycz et al. 2025; Balan et al. 2025). In addition, existing literature is often fragmented across feed types, production systems, and regions, limiting the development of a cohesive and updated perspective.

**Fig. 1:**
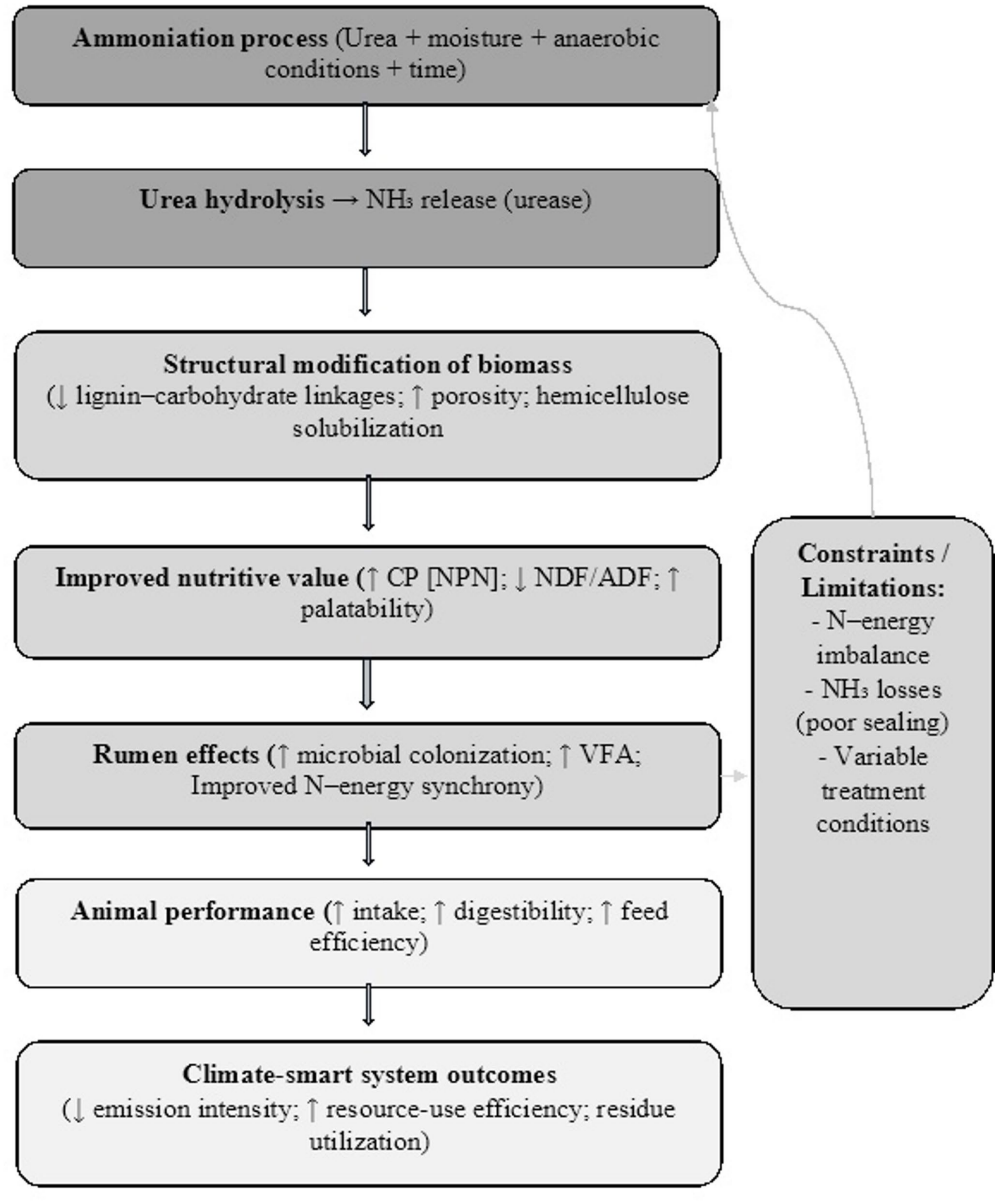
Provides a schematic representation of the ammoniation process, highlighting urea hydrolysis, ammonia-induced cleavage of lignin–carbohydrate linkages, hemicellulose solubilization, and fiber swelling, which collectively contribute to increased cell wall porosity and enhanced microbial accessibility in the rumen

This review therefore provides a comprehensive synthesis of findings published between 2015 and 2025, while incorporating foundational studies where necessary. It examines ammoniation mechanisms, feedstock-specific responses, rumen fermentation dynamics, digestibility, and animal performance, and further discusses emerging environmental and climate-smart implications, practical considerations, and adoption pathways in tropical and semi-arid livestock systems. These interconnected processes form the basis for evaluating ammoniation not only as a feed improvement strategy but also as a system-level intervention influencing productivity and environmental performance. A conceptual overview of the ammoniation process and its effects on roughage quality, rumen function, and animal performance is presented in Fig. 1.

## Literature search strategy

A structured literature search was conducted using major scientific databases (Scopus, Web of Science, PubMed, and Google Scholar) to identify peer-reviewed articles relevant to crop residue treatment, ammoniation, rumen function, and ruminant performance in tropical and semi-arid systems. Search terms included combinations of keywords such as “ammoniation”, “urea treatment”, “crop residues”, “ruminant nutrition”, “digestibility”, and “tropical livestock”. Priority was given to studies published in the last decade, while earlier seminal works were included where necessary to provide context.

Older foundational studies were retained where they provide essential mechanistic insights or widely accepted baseline values for ammoniation responses. Reference lists of selected articles were also screened to capture additional relevant studies. Data presented in Tables 1, 2, 3, 4, 5, 6A, 6B and 7 were synthesized from ranges and consistent trends reported across selected studies, rather than from formal meta-analysis. Only studies with comparable treatment conditions (e.g., 3–5% urea, adequate moisture, and anaerobic storage) were considered to ensure consistency.

**Table 1:** Effects of ammoniation on chemical composition of low-quality roughages (synthesized from published studies)

Parameter	Untreated roughages	Ammoniated roughages	Typical response to ammoniation
Dry matter (%)	94–96	94–96	Largely unchanged
CP (% DM)	4–6	9–12	Increased 40–100% due to N incorporation
Ash (% DM)	12–14	13–15	Slight increase or variable
NDF (% DM)	64–68	58–63	Reduced 5–12%
ADF (% DM)	50–55	47–50	Reduced 5–10%
ADL (% DM)	1.2–1.4	1.2–1.4	Minimal change

Note: Values are ranges synthesized from peer-reviewed studies under comparable ammoniation conditions (typically 3–5% urea, adequate moisture, and anaerobic storage), including Ma et al. (2020), Carvalho et al. (2024) and Rashid et al. (2025). Variability reflects differences in feedstock, treatment conditions, and lignocellulosic characteristics

**Table 2:** Effects of ammoniation on rumen fermentation characteristics of low-quality roughages

Fermentation parameter	Untreated roughages	Ammoniated roughages	Typical response to ammoniation
Total VFA (mmol/L)	60–70	75–90	Increased 10–30% due to improved fermentability
Acetate (mmol/L)	40–45	48–55	Increase reflects enhanced fiber digestion
Propionate (mmol/L)	18–20	20–23	Improved energy efficiency
Butyrate (mmol/L)	5–6	6–7	Moderate increase
Rumen pH	6.7–6.9	6.7–6.9	Generally unchanged, remains optimal

Note: Values represent ranges synthesized from multiple peer-reviewed studies conducted under comparable ammoniation conditions (typically 3–5% urea, adequate moisture, and anaerobic storage). Data are derived from consistent trends reported across studies, including Ma et al. (2020), Carvalho et al. (2024), and Rashid et al. (2025). Variability reflects differences in feedstock type, treatment conditions, experimental design, and between in vitro and in vivo systems

**Table 3:** Generalized effects of ammoniation on major cereal crop residues used in ruminant feeding

Feedstock	CP response	NDF/ADF response	Digestibility response	Strength of response	Key references
Rice straw	Substantial increase (2×)	Moderate reduction	Moderate to high increase	Strong	Ma et al. (2020); Li et al. (2020)
Wheat straw	Clear increase	Slight to moderate reduction	Moderate increase	Moderate	Rashid et al. (2025); Matthews et al. (2019)
Maize stover	Clear increase	Moderate reduction	High increase	Strong	Carvalho et al. (2024); Kamal et al. (2025)
Sorghum stover	Moderate increase	Variable reduction	Moderate increase	Moderate	Shah et al. (2025); Kamal et al. (2025)

Note: Responses are generalized from multiple peer-reviewed studies under typical ammoniation conditions (3–5% urea, adequate moisture, and anaerobic storage). Strength of response is qualitatively classified based on the magnitude and consistency of reported effects across studies. Variability reflects differences in feedstock characteristics, treatment conditions, and stage of maturity

**Table 4:** Effects of ammoniated roughage inclusion on intake and nutrient digestibility in growing ruminants (synthesized from published studies)

Parameter	Diets without ammoniated roughage	Diets with ammoniated roughage	Typical response to ammoniation
Dry matter intake (% BW)	~ 1.8–2.2	~ 2.2–2.8	Increased by 10–40% due to improved palatability and reduced rumen fill limitation
Organic matter intake	Lower (due to lower DMI)	Higher (reflecting increased DMI)	Increases proportionally with DMI and dietary composition
CPI	—	—	Increased due to higher DMI and additional NPN
NDFI	—	—	Reflects increased DMI despite improved fiber degradability
DMD (%)	68–72	74–78	Increased by 5–10% units
Organic matter digestibility (%)	72–75	77–82	Consistent improvement across studies
CPD (%)	78–80	83–87	Improved nitrogen utilization
NDFD (%)	50–53	56–60	Enhanced fiber degradation due to cell wall disruption

Note: Values represent ranges synthesized from peer-reviewed studies under comparable ammoniation conditions (typically 3–5% urea, adequate moisture, and anaerobic storage), including Rashid et al. (2025), Carvalho et al. (2024), and Ribeiro et al. (2020). Organic matter, crude protein, and fiber intake were not independently quantified, as they are directly derived from dry matter intake (DMI) and diet composition; responses are therefore interpreted relative to changes in DMI. CPI = crude protein intake; NDFI = neutral detergent fiber intake; DMD = dry matter digestibility; CPD = crude protein digestibility; NDFD = neutral detergent fiber digestibility. Variability reflects differences in diet composition, inclusion level, and animal species

**Table 5:** Effects of ammoniation on in vitro dry matter and fiber digestibility of low-quality roughages

Digestibility parameter	Untreated roughages	Ammoniated roughages	Typical response to ammoniation
In vitro DM digestibility (%)	50–55	60–70	Increased by 8–20% units
In vitro NDF digestibility (%)	40–45	50–65	Improved by 10–25% units

Note: Values are ranges synthesized from peer-reviewed studies under comparable ammoniation conditions (typically 3–5% urea, adequate moisture, and anaerobic storage), including Ma et al. (2020), Hemati et al. (2022), and Rashid et al. (2025). Variability reflects differences in roughage type, treatment conditions, and in vitro incubation protocols

**Table 6A:** Effects of ammoniation of low-quality roughages on intake and growth performance in ruminants (synthesized from published studies)

Species	Feed type	Treatment	DMI (kg/d)	FBW (kg)	ADG (kg/d)	Reference
Lambs	Maize stover	0 vs. 10–40%	0.22–0.25	33.93–39.20	0.18–0.25	Chelopo and Marume (2022)
Goats	Cowpea husk	0 vs. 350–700 g/kg	0.562–0.575	—	0.068–0.079	Olafadehan et al. (2022)
Cattle	Tropical grass hay	Ammoniated ± supplementation	—	—	0.49–0.85	Brown and Johnson (1991)
Buffalo calves	Wheat straw	Control vs. 0–100%	—	106.0–117.5	—*	Rashid et al. (2025)

Notes: DMI = dry matter intake; FBW = final body weight; ADG = average daily gain; supp. = supplementation. Treatment values indicate inclusion levels or comparisons between untreated (control) and ammoniated roughages on a dry matter basis. Missing values (—) indicate parameters not reported. In Rashid et al. (2025), body weight gain (BWG: 35.8–47.1 kg) was reported instead of ADG. Older foundational studies (e.g., Brown and Johnson 1991) were included where recent comparable data were limited. Overall, ammoniation is associated with increased intake and improved growth performance, with responses influenced by inclusion level, diet composition, and species

**Table 6B:** Effects of ammoniation of low-quality roughages on feed efficiency and performance outcomes in ruminants (synthesized from published studies)

Species	FCR	Performance response
Lambs	5.25–6.20	Optimal inclusion (10–20%) improved FE; higher levels reduced efficiency
Goats	7.24–8.44	Moderate inclusion improved FE and growth; intake unchanged
Cattle	—	FE not reported; intake and digestibility improvements supported growth
Buffalo calves	7.2–7.7	Growth improved; slight reduction in FE at high inclusion

Note: FCR = feed conversion ratio (kg feed/kg gain); lower values indicate greater efficiency. Values are synthesized from peer-reviewed studies evaluating ammoniated roughages under varying inclusion levels. Missing values (—) indicate parameters not reported. Variability reflects differences in diet composition, inclusion level, and animal species

**Table 7:** Regional barriers and enabling factors influencing adoption of ammoniation technology in tropical livestock systems (synthesized from published literature)

Region	Key barriers to adoption	Major enabling factors
South Asia	Labor-intensive handling; inconsistent urea supply; safety concerns	Strong government extension services; farmer demonstrations; high demand for dry-season feed
East Africa	Limited airtight storage facilities; weak extension support; low technical skills	NGO and cooperative involvement; communal treatment units; increasing awareness
West Africa	Urea price fluctuations; low farmer awareness	Abundant crop residues; growing interest in dry-season feeding strategies
China	Labor requirements; limited pit or storage space	Long-standing experience with ammoniation; reliable urea availability; strong research and extension networks
Latin America	Competing feed alternatives; limited labor availability	Climate-smart livestock initiatives; large volumes of crop residues
Middle East / Central Asia	Water scarcity; limited technical guidance	High availability of cereal straw; need for low-cost feeding options

Note: Authors’ synthesis based on published literature (Adesogan et al. 2025; Ondiek et al. 2025; Azumah and Adzawla 2017; de Moraes et al. 2023; Kumssa 2017). NGO: Non-Governmental Organization

### Mechanisms of ammoniation

Ammoniation modifies lignocellulosic materials through the release of ammonia, which penetrates plant tissues and disrupts lignin–carbohydrate linkages, improving the accessibility of structural carbohydrates to rumen microorganisms (Fan et al. 2024; Carvalho et al. 2024). Urea hydrolysis, facilitated by moisture and urease activity, generates ammonia under anaerobic conditions, leading to increased pH and partial solubilization of hemicellulose (Fan et al. 2024). The equilibrium between ammonia (NH₃) and ammonium (NH₄⁺) is pH-dependent, with alkaline conditions (pH 8–9) favoring free ammonia (NH₃), the more reactive species responsible for cleaving lignin–carbohydrate linkages. Structural changes, including increased porosity and reduced fiber rigidity, have been confirmed using analytical techniques such as Fourier Transform Infrared Spectroscopy (FTIR) and Scanning Electron Microscopy (SEM) and are associated with enhanced microbial attachment and enzymatic degradation (Li et al. 2020; Weimer 2022).

The efficiency of these processes depends on treatment conditions, particularly moisture, temperature, and pH, which influence urea hydrolysis and ammonia diffusion within the biomass (Lei et al. 2018; Bai et al. 2025). In tropical environments, higher temperatures generally accelerate ammonia release, while adequate moisture ensures effective penetration. These factors interact with pH to regulate the balance between NH₃ and NH₄⁺, thereby influencing the efficiency of structural disruption and nitrogen fixation within the treated biomass.

Ammoniation is governed not only by chemical interactions but also by reaction kinetics influenced by environmental conditions. Urea hydrolysis, catalyzed by urease, generally follows first-order kinetics under field conditions, with the rate strongly dependent on temperature and moisture availability (Bai et al. 2025). In tropical environments, elevated temperatures (30–40 °C) accelerate urea hydrolysis and ammonia release, thereby reducing the time required for effective treatment compared to cooler conditions (Lei et al. 2018; Bai et al. 2025). Moisture content (typically 30–50%) is critical for facilitating urea dissolution and enzymatic activity, while also enhancing ammonia diffusion within the biomass matrix.

From a thermodynamic perspective, the equilibrium between ammonia (NH₃) and ammonium (NH₄⁺) is pH-dependent, with alkaline conditions favoring NH₃, the more reactive form responsible for lignin–carbohydrate bond cleavage (Impraim et al. 2023). The efficiency of nitrogen fixation within the treated material therefore reflects the combined effects of reaction rate, ammonia availability, and diffusion dynamics (Muzzo 2026). These interacting factors ultimately determine the extent of structural modification and improvement in digestibility observed in ammoniated roughages.

## Effects of ammoniation on roughage characteristics

### Chemical composition

Ammoniation increases CP content through the incorporation of non-protein nitrogen (NPN) and decreases neutral detergent fiber (NDF) and acid detergent fiber (ADF) via partial cleavage of lignin–carbohydrate bonds and solubilization of hemicellulose (Ma et al. 2020). Across studies, CP typically increases from 5 to 6% to 9–12%, under standard ammoniation conditions while NDF and ADF decrease by 5–12%, improving rumen fermentation efficiency and digestibility. These changes are driven by ammonia-induced disruption of ester linkages within the cell wall matrix, enhancing the accessibility of structural carbohydrates to microbial enzymes (Carvalho et al. 2024; Li et al. 2020).

However, the magnitude of these changes varies among crop residues, with cereal straws differing in response depending on lignin and silica content (Van Soest et al. 1984; Carvalho et al. 2024). In addition, the increase in CP largely reflects NPN, which requires adequate fermentable energy for efficient microbial utilization (Van Soest 1994). These compositional changes are summarized in Table 1, while Table 3 presents a synthesized comparison across selected cereal-based crop residues commonly used in ruminant feeding systems. This table provides a qualitative synthesis of consistent trends reported across studies, complementing the quantitative ranges presented in Table 1.

### Feedstock-dependent variation in response to ammoniation

Rice straw typically exhibits a more limited response to ammoniation due to its high silica content, which restricts ammonia penetration and reduces the extent of cell wall disruption (Agbagla-Dohnani et al. 2012; Datsomor et al. 2022). In contrast, maize stover, with comparatively lower silica and moderate lignin content, generally shows greater improvements in fiber degradability and digestibility (Atuhaire et al. 2016). Mature tropical grasses, depending on their stage of maturity and lignification, often display variable responses, with more pronounced improvements observed in highly lignified materials where structural disruption significantly enhances microbial accessibility (Bichot et al. 2018). These differences highlight the importance of feedstock selection and characterization in optimizing ammoniation efficiency across production systems, as intrinsic properties such as lignin concentration, silica content, and cell-wall architecture ultimately determine the extent of structural modification and digestibility improvement (Bichot et al. 2018; Jayasinghe et al. 2022).

### Rumen fermentation characteristics

Ammoniation enhances rumen fermentability, as reflected in increased concentrations of volatile fatty acids (VFA), particularly acetate, propionate, and butyrate (Rashid et al. 2025). This increase improves metabolizable energy availability for meat, milk, and growth. Across studies, treated roughages commonly show 10–30% higher total VFA production while maintaining a stable rumen pH (6.7–6.8) (Paswan et al. 2022).

In addition, ammoniation provides non-protein nitrogen (NPN), which can support microbial activity under adequate fermentable energy conditions (Niazifar et al. 2024; Weimer 2022). However, fermentation responses are influenced by overall diet composition, particularly the balance between nitrogen supply and available carbohydrates (Kand et al. 2018). Rumen fermentation responses to ammoniated roughages are summarized in Table 2.

### Synchrony of nitrogen and energy in the rumen

Ammoniation increases crude protein (CP) content primarily through the incorporation of non-protein nitrogen (NPN), which is rapidly hydrolyzed to ammonia in the rumen (Carvalho et al. 2024). The efficient utilization of this ammonia depends on its synchronization with the availability of fermentable carbohydrates, which provide the energy required for microbial protein synthesis.

When the rate of ammonia release exceeds the availability of fermentable energy, nitrogen capture by rumen microorganisms is reduced, leading to increased ruminal ammonia accumulation and subsequent nitrogen losses (Kand et al. 2018; Ribeiro et al. 2020; Nichols et al. 2022). Conversely, improved synchrony enhances microbial growth efficiency and maximizes the conversion of ammonia into microbial protein.

Therefore, balancing ammoniated roughages with appropriate energy sources is essential to optimize nitrogen use efficiency, support microbial protein synthesis, and improve overall animal performance.

### Physical and sensory qualities

Ammoniated feeds become softer, less brittle, more elastic, and more palatable because ammonia disrupts fiber rigidity (Li et al. 2020). They also develop a characteristic ammonia odor that generally does not reduce intake. High dietary NDF typically restricts intake by increasing rumen fill; however, fiber swelling and reduced rigidity in ammoniated roughages promote intake, digestion, and overall animal performance (Koppel et al. 2015; Siele 2019). Nevertheless, excessively treated or poorly aerated materials may retain strong ammonia odors, which can temporarily depress intake if not adequately ventilated prior to feeding (Li et al. 2020). Improvements in feed intake and utilization parameters following ammoniation of low-quality roughages are presented in Table 4.

### Toxicological considerations

Ammoniation of roughages is generally safe when properly managed; however, under certain conditions, undesirable compounds such as imidazoles (e.g., 4-methylimidazole) may be formed, particularly when treating high-sugar materials or when excessive heating occurs during the process (Al-Shathry 2003; Müller et al. 1998). These compounds have been associated with abnormal neurological symptoms in ruminants (bovine bonkers syndrome), although such cases are rare and typically linked to improper treatment conditions (Niles 2017; Washburn 2017). Risk factors include high soluble carbohydrate content, inadequate aeration, excessive ammonia concentration, and prolonged storage under unfavorable conditions (Leng 1990). To mitigate these risks, it is essential to ensure appropriate urea inclusion rates, adequate moisture, proper sealing during treatment, and sufficient aeration before feeding (Huss et al. 2018). When these guidelines are followed, ammoniation remains a safe and effective method for improving roughage quality.

### Digestibility and rumen degradation

Ammoniation improves in vivo and in vitro digestibility by 5–20% units, depending on roughage type and treatment conditions (Matthews et al. 2019). These improvements are largely attributed to enhanced accessibility of structural carbohydrates and increased availability of fermentable substrates (Khan and Ahring 2019; Hemati et al. 2022). Enhanced digestibility is further supported by more efficient utilization of NPN, provided adequate fermentable energy is available for microbial growth (Ye et al. 2025; Zurak et al. 2023). Consequently, ammoniated roughages exhibit higher in vitro dry matter digestibility (IVDMD) and NDF digestibility, as well as increased in vivo digestibility of dry matter (DM), organic matter (OM), and CP (Sanders 2024; Cherdthong and Wanapat 2013). The magnitude of response varies with feedstock characteristics and treatment conditions, with more pronounced improvements in highly lignified materials (Main et al. 2025). In vitro digestibility responses are presented in Table 5 (Rashid et al. 2025).

### Synergistic treatment approaches

Recent research has explored the integration of ammoniation with complementary treatment strategies to further enhance the nutritive value of low-quality roughages. In particular, the combined use of ammoniation with exogenous fibrolytic enzymes or biological treatments such as white-rot fungi has shown promising results (Datsomor et al. 2022; Hu et al. 2021). Ammoniation disrupts lignocellulosic structures and increases fiber accessibility, thereby enhancing the effectiveness of enzymes that degrade cellulose and hemicellulose (Leon-Tinoco et al. 2025; Terry et al. 2020). Similarly, fungal treatments can selectively degrade lignin and improve fiber digestibility when applied sequentially or in combination with ammoniation (Hu et al. 2021).

These synergistic approaches have been associated with improved nutrient availability, increased digestibility, and enhanced animal performance compared to single-treatment methods (Mor et al. 2018). However, their adoption remains limited due to higher costs, longer processing times, and the need for controlled treatment conditions. Further research is required to optimize these integrated systems under practical field conditions, particularly in tropical smallholder production systems, where cost and operational complexity continue to limit their scalability.

### Animal performance response

Ammoniation improves animal performance through enhanced nutrient availability and more efficient rumen utilization, resulting in increased feed intake, growth rate, and feed efficiency (FE) (Rashid et al. 2025; Carvalho et al. 2024). Feed intake generally increases by 10–40%, contributing to improved daily weight gain and final body weight.

Studies in cattle and buffalo calves show that increasing inclusion of ammoniated roughages generally improves growth performance and nutrient utilization (Mor et al. 2018). However, responses are not always linear, as moderate inclusion levels tend to optimize performance, while excessive inclusion may reduce efficiency due to imbalances between nitrogen supply and available fermentable energy (Arias et al. 2020). The magnitude of response depends on diet composition, supplementation level, and animal physiological status (Bodine et al. 2001). Intake and growth performance responses are summarized in Table 6A, while FE and associated performance outcomes are presented in Table 6B.

Improvements in intake and growth performance observed across studies (Table 6A) are generally associated with enhanced FE outcomes (Table 6B), reflecting improved utilization of structural carbohydrates and more effective capture of ammonia-derived nitrogen in the rumen (Carvalho et al. 2024; Ribeiro et al. 2020). These responses highlight the importance of synchronizing nitrogen release with available fermentable energy to maximize microbial protein synthesis and overall feed efficiency (Kand et al. 2018; Ribeiro et al. 2020).

Beyond productivity gains, these improvements in FE have important implications at the production-system level, particularly in terms of emission intensity and resource-use efficiency (Moorby and Fraser 2021). However, the variability in reported responses across studies suggests that ammoniation alone is not sufficient to guarantee improved performance, and its effectiveness depends strongly on diet formulation, particularly the balance between nitrogen supply and fermentable energy (Bezerra et al. 2025).

### Environmental and climate-smart implications of ammoniation

Building on the improvements in digestibility, intake, and animal performance described above, ammoniation also influences environmental outcomes at the system level. From a life cycle assessment (LCA) perspective, ammoniation can contribute to reducing the environmental footprint of ruminant production, particularly in terms of emission intensity (i.e., greenhouse gas emissions per unit of animal product) (Boyce et al. 2024; O’Brien et al. 2016). Improvements in fiber digestibility and feed conversion efficiency reduce the proportion of dietary energy lost as enteric methane, as more digestible diets shift rumen fermentation toward more efficient pathways and increase animal productivity per unit of feed consumed (Santander et al. 2023; Weimer 2022).

Although absolute methane emissions per animal may not always decrease, several studies indicate that improved FE can lower methane emissions per unit of milk or live weight gain by approximately 10–20%, depending on diet composition and production system (Løvendahl et al. 2018). In addition, ammoniation reduces the need for external concentrate feeds and discourages open-field burning of crop residues, thereby lowering upstream and indirect emissions. However, the overall environmental benefit depends on proper management of ammonia to minimize nitrogen losses through volatilization, which can offset some of the gains if poorly controlled (O’Brien et al. 2016). Integrating ammoniation into climate-smart livestock systems therefore requires a balance between improved feed utilization, emission intensity reduction, and efficient nitrogen management across the production chain.

### Practical and economic considerations

Urea ammoniation is a relatively cost-effective technology that relies on readily available inputs such as urea, water, and simple storage structures. It reduces reliance on expensive concentrates and improves the utilization of crop residues, making it particularly suitable for smallholder production systems (Kumssa 2017). Community- or cooperative-based treatment approaches can further reduce labor requirements and enhance adoption by lowering per-unit treatment costs. From a sustainability perspective, ammoniation contributes to improved resource use efficiency by converting low-quality residues into valuable feed resources, thereby reducing the need for external feed inputs (Kamal et al. 2025; de Moraes et al. 2023). In addition, improved feed utilization may lower emission intensity (e.g., methane per unit of product) through enhanced animal productivity (Adesogan et al. 2025). However, adoption may be constrained by factors such as labor requirements, need for proper handling of urea, variability in treatment conditions, and limited technical knowledge among smallholders (Kumssa 2017). These constraints highlight the importance of extension support and appropriate management practices for successful implementation.

### Digital tools and precision support for ammoniation

Recent advances in digital agriculture (Agriculture 4.0) offer new opportunities to improve the accuracy and adoption of ammoniation technology, particularly among smallholder farmers (Mihret et al. 2025). Mobile-based decision-support tools and advisory platforms can assist in calculating appropriate urea inclusion rates, moisture levels, and treatment duration based on local conditions and feedstock type. In addition, low-cost sensors and simple monitoring devices can help estimate moisture content and ensure adequate sealing, thereby reducing variability in treatment outcomes and minimizing ammonia losses through volatilization (Anas et al. 2025; Joo et al. 2023). Digital extension services, including smartphone applications and SMS-based advisory systems, can further enhance knowledge transfer, provide real-time guidance, and reduce human error during the treatment process (Tadele et al. 2025). Although still emerging in many tropical livestock systems, the integration of such tools has the potential to improve the consistency, safety, and scalability of ammoniation as a climate-smart feeding strategy.

### Global adoption of ammoniation

Ammoniation has been promoted and adopted to varying degrees across Southeast Asia, South Asia, East and West Africa, and parts of Latin America, largely due to the abundant availability of low-quality crop residues, pronounced dry-season feed shortages, and institutional support from governments and non-governmental organizations. In countries such as Indonesia, India, Bangladesh, China, and Vietnam, ammoniation has contributed to improving the nutritive value of rice straw and maize stover, with reported increases in CP content of 30–70% and improvements in digestibility of 10–20% (Pamungkas et al. 2025; Li et al. 2020).

However, the level of adoption remains heterogeneous, with uptake often limited by labor requirements, input accessibility, technical knowledge, and consistency of treatment conditions. As a result, while ammoniation is well established in research and development programs, its practical application at farm level varies widely across regions. Regional adoption patterns, enabling factors, and constraints associated with ammoniation technology are presented in Table 7.

### Limitations of ammoniation

Adoption of ammoniation technology is constrained by several practical and institutional factors, including labor-intensive handling, the need for airtight storage conditions (Bello and Abdulwasiu 2026), risks of urea toxicity when treatment is poorly managed, fluctuations in urea prices, and the technical skill required to achieve optimal moisture levels and effective sealing (Bezerra et al. 2025). In addition, environmental conditions such as excessive humidity or inadequate sealing can result in uneven treatment, mold development, and ammonia losses, thereby reducing treatment efficiency and feed quality (Whitlow and Hagler 2010).

These limitations are particularly pronounced in smallholder systems with limited access to extension services, training, and inputs, which can hinder consistent and safe application of the technology (Pamungkas et al. 2025; Azumah and Adzawla 2017). Addressing these constraints through improved extension support, training, and simple decision-support tools is essential to enhance adoption and ensure effective implementation under field conditions.

## Conclusion

Ammoniation of low-quality roughages remains a practical and cost-effective strategy for improving feed quality and sustaining ruminant productivity in tropical and semi-arid regions. Its effectiveness is primarily driven by ammonia-induced modification of lignocellulosic structures and increased nitrogen availability, which enhance rumen fermentation, nutrient utilization, and animal performance. At the system level, ammoniation contributes to climate-smart livestock production by improving feed efficiency (FE) and reducing emission intensity per unit of output. However, these benefits depend on proper synchronization of nitrogen and energy in the rumen, as well as effective control of nitrogen losses during treatment.

Emerging strategies, including integration with biological or enzymatic treatments and the use of digital decision-support tools, offer opportunities to improve treatment efficiency, consistency, and adoption. Nevertheless, wider implementation remains constrained by labor requirements, input accessibility, and technical capacity. Future research should focus on optimizing treatment conditions across diverse production environments, quantifying environmental impacts through life cycle assessment, and developing scalable technologies to support practical application. Overall, ammoniation represents a viable pathway for enhancing feed utilization, reducing environmental impact, and strengthening the resilience of ruminant production systems.

### Statement of originality

This manuscript has not been previously published and is not under consideration for publication elsewhere.

## Data Availability

Not applicable.
